# Upregulation of KHDC1L promotes the proliferation and inhibits apoptosis in head and neck squamous cell carcinoma

**DOI:** 10.1080/15592294.2023.2175168

**Published:** 2023-02-03

**Authors:** Qi Zhang, Shuimei Luo, Yang Luo, Yiqiang Huang, Ziming Wang, Xianhe Xie

**Affiliations:** a Department of Oncology, Molecular Oncology Research Institute, The First Affiliated Hospital, Fujian Medical University, Fuzhou, Fujian 350000, China; b Department of Oncology, National Regional Medical Center, Binhai Campus of the First Affiliated Hospital, Fujian Medical University, Fuzhou 350212, China; c Fujian Key Laboratory of Precision Medicine for Cancer, The First Affiliated Hospital, Fujian Medical University, Fuzhou, Fujian 350000, China

**Keywords:** KHDC1L, head and neck squamous cell carcinoma, proliferation, apoptosis, AKT

## Abstract

Head and neck squamous cell carcinoma (HNSCC) remains a dreadful malignancy bearing poor clinical efficacy, with emerging evidences indicating RNA-binding proteins’ (RBPs’) relevance to the evolution of the disease. Categorized as RBPs, the K-homology domain-containing 1 (KHDC1) family is proved to be closely related to cell survival and death. As a novel KHDC1 member, only one study is currently available in osteoarthritis synovial cells to unveil KHDC1L’s function of promoting proliferation. Nevertheless, to the best of our knowledge, the role of KHDC1L in human tumour is yet to be fully explored. On the basis of The Cancer Genome Atlas (TCGA) database and cell lines comparison with normal counterparts in this study, we first discovered *KHDC1L* to be overexpressed in HNSCC. According to bioinformatics analysis, apoptosis and P53 pathways were remarkably enriched in the *KHDC1L* low-expression group in TCGA database. Moreover, in vitro experiments were applied to verify that upregulation of KHDC1L could promote the proliferation and inhibit apoptosis in HNSCC cells CAL27. Transcriptome sequencing ascertained downstream differentially expressed genes to be significantly enriched in PI3K-AKT pathways. Furthermore, as validated by western blot, we found an elevated expression level of pAKT/AKT and Bcl-2, constant expression level of BAX, together with decreased activity of Caspase-3 and PARP-1 in the KHDC1L-upregulated group. In conclusion, our study pioneeringly elaborated that KHDC1L could promote proliferation and inhibit apoptosis in HNSCC cell CAL27 via AKT and Bcl-2 pathways, representing a crucial step for seeking a new diagnostic and therapeutic target in HNSCC.

## Introduction

More than 830,000 cases of head and neck cancers are annually diagnosed all over the world, with over 430,000 deaths each year [1]. The disease mainly involves heterogeneous malignances arising from upper aerodigestive tract [2], 90% of which are head and neck squamous cell carcinoma (HNSCC) [3]. Various clinical outcomes in oral cancer may occur as affected by distinct molecular characteristics [4]. Moreover, the prognosis remains unsatisfying [5,6] in general although recent therapeutic modality has been substantially improved. Therefore, further researches on the progression of HNSCC are urgently needed to clarify the mechanism and identify effective therapeutic target.

RNA binding proteins (RBPs) orchestrate several cellular activities at post-transcriptional level involving RNA stability and translation [7]. More than 1,500 proteins were annotated as RBPs [8], in which alterations may cause numerous diseases due to its critical role in basic cellular processes [9]. In addition, RBPs contribute to tumorigenesis and progression [10] based on current studies. As to HNSCC, loss function of HuR alters the oncogenic characteristics through effecting downstream target mRNAs expression levels [11]. Moreover, in hyopharyngeal cancer, knockdown of RBP IGF2BP2 was proved to inhibit proliferation and promote apoptosis in vitro and vivo [12]. In conclusion, a comprehensive exploration of RBPs will not only contribute to further study of the pathogenesis but also identify novel markers for HNSCC detection as a whole.

Recently, members of the K-homology domain-containing 1 (KHDC1) family were identified as RBPs. In a study of Nur77-induced apoptosis in T cells, *Khdc1a* served as its downstream novel transcript, and overexpression of KHDC1A induced apoptosis in vitro and in vivo [13,14]. The *KHDC1* family includes KH domain-containing 1 like (*KHDC1L*) gene on chromosome 6q13, and the protein primary sequence of KHDC1L with 128 amino acids enjoys 84.4% similarity to KHDC1 as presented in the cytoplasm. KHDC1L expression was elevated in synovial tissue of patients with osteoarthritis, and downregulation of its expression inhibited synovial cell proliferation [15]. These findings figured out RBPs KHDC1 family to be closely related to survival signalling, with its specific role in cancer yet to be explored.

In the present study, we first found *KHDC1L* to be highly expressed in HNSCC tissues and cell lines, whose overexpression could influence the HNSCC cell growth and apoptosis in vitro. Moreover, by means of transcriptome sequencing and western blot, this study discovered that AKT and Bcl-2 might serve as KHDC1L downstream pathway. In summary, our study took the lead in identifying KHDC1L as a novel molecular biomarker in HNSCC, contributing to an effective target against cancer.

## Materials and methods

### Cell culture

The human oral epithelial cell line HOK was purchased from WHELAB Bioscience Limited (Shanghai, China), the HNSCC cell lines SCC9 and CAL27 were acquired from MeisenCTCC (Meisen Chinese Tissue Culture Collections, Jinhua, China) and Procell Technology Co., Ltd. (Wuhan, China). Furthermore, HOK cells were cultured in MEM (Invitrogen, Carlsbad, CA, USA), while SCC9 and CAL27 cells were in DMEM (Invitrogen), with all medium containing 10% FBS (HyClone, Logan, UT, USA). Identified by STR analysis, all the cells were grown in an incubator with 37°C, 5% CO2.

### RNA extraction and real-time PCR

TransZol Up Plus RNA Kit (TransGen Biotech, Beijing, China) was utilized to extract total RNA, and the cDNA was synthesized with Superscript Reverse Transcriptase Kit (TransGen Biotech). In addition, ChamQ Universal SYBR qPCR Master Mix (Vazyme, Nanjing, China) was applied to perform qPCR, via Applied Biosystems 7500 Real-Time PCR Systems. Meanwhile, the data were normalized to GAPDH mRNA, with qPCR primers for KHDC1L as follows: forward 5′- GACTTCATGACACGTACCTTCG-3′ and reverse 5′-AGCGTGACACTTGGAGTCCT-3′.

### Vector construction and transfection

The synthesis of vector plasmid pcDNA3.1-KHDC1L containing the human KHDC1L gene sequence with 3× flag tag and pcDNA3.1 were commissioned by HANBIO (Shanghai, China). Furthermore, CAL27 cells with a 70–80% confluence were transfected with plasmid (pcDNA3.1-KHDC1L or pcDNA3.1) (3ug per well) in six-well plate via GP-transfect-mate (GenePharma, Suzhou, China) following the manufacturer’s instruction.

### Transcriptome sequencing and bioinformatics analysis

The total RNA of overexpressed group and the control group of CAL27 cells was extracted after lysis and sent to BGI Biologics Co., Ltd, where qualified RNA was selected for transcriptome sequencing. Moreover, bioinformatics analysis on transcriptome sequencing data was completed based on BGI Gene Sequencing Analysis Platform (https://biosys.bgi.com/), including differentially expressed gene analysis, Kyoto Encyclopedia of Genes and Genomes (KEGG) enrichment analysis, Gene Ontology (GO) enrichment analysis, Gene Set enrichment analysis (GSEA), as well as protein–protein interaction (PPI) network analysis.

### Western blot analysis

In this study, cells were incubated in RIPA buffer (#P0013B; Beyotime, Shanghai, China) containing protease inhibitor (#K1007; APExBIO, Houston, America) and phosphatase inhibitor (#K1015; APExBIO) at 4°C. After 30 min, lysates were centrifuged at 12,000 rpm for 15 min at 4°C to obtain the supernatant. Proteins were quantified with a BCA assay kit (#P0010S, Beyotime), and 20 μg per lane were separated by SDS-PAGE and transferred to PVDF membranes (#IPVH00010, Millipore, Shanghai, America). Then, the membrane was incubated for 1 h at room temperature with HRP-conjugated antibody and developed using ECL reagent (#P0018S, Beyotime). Furthermore, antibodies against flag Tag (#8146, 1:1000), phospho-AKT (Ser473) (#13038, 1:1000), AKT (#4691, 1:3000), Bcl-2 (#15071, 1:1000), BAX (#5023, 1:1000) and cleaved Caspase-3 (#9661, 1:3000) were ordered from Cell Signaling Technology (Cell Signaling Technology, Danvers, MA). Besides, cleaved PARP-1 (#ab32064, 1:1000) was purchased from Abcam (Abcam, Cambridge, UK), antibodies against β-actin (#YT0099, 1:3000) and HRP Goat anti-mouse IgG(H + L) (#RS0001,1:5000) from ImmunoWay (Jiangsu, China) and anti-rabbit IgG HRP-linked antibody (#7074, 1:5000) from Cell Signaling Technology.

### Cell morphology, CCK8 assays, cell counting assays, and colony assays

Cell morphology was observed under the microscope and photographed after transfection, with a CCK-8 assay kit (Abmole Bioscience, Shanghai, China) to measure viability. First, CAL27 cells were seeded into 96-well plates and incubated for the indicated times. Then, 10 µl of CCK-8 solution was added and incubated at 37°C for 1 h. Cells were seeded in 12-well plates after measuring the absorbance at 450 nm. After 4 d of transfection, they were trypsinized, resuspended in the culture medium, put onto haemocytometer to count numbers under the microscope. Furthermore, single-cell suspensions of CAL27 cells were seeded into six-well plates at 103 cells/well and incubated for 14 d. Then, the cells were finally counted.

### Flow cytometry assays

Apoptotic cells were detected by Annexin V-FITC/PI Apoptosis Kit I (#KGA107; KeyGEN BioTECH, Jiangsu, China). In brief, cells were trypsinized, washed twice with the phosphate-buffered saline, and resuspended in 1× binding buffer. By means of a flow cytometer (BD Accuri C6, America), 100 μL of the solution was supplemented with 5 μL of AnnexinV-FITC as well as propidium iodide, and incubated for 15 min at room temperature in the dark.

### Statistical analysis

Based on Prism version 5.0 (GraphPad Software, San Diego, CA, USA), data were analysed via Student’s *t* test, one-way ANOVA, two-way ANOVA and log-rank test. In addition, the mean and standard deviation (SD) are determined in accordance with at least three independent experiments, regarding *P* < 0.05 as statistically significant

## Results

### KHDC1L *is aberrantly highly expressed in HNSCC*

**Figure 1.**
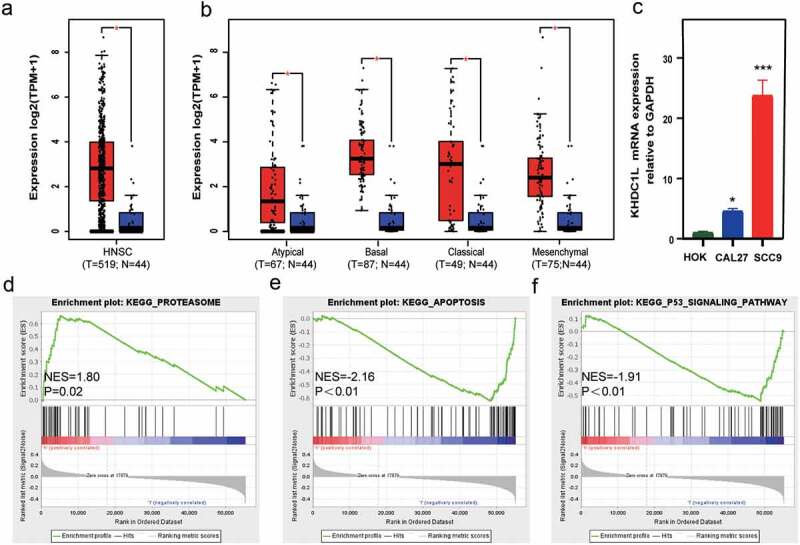
Integrated analysis on the expression and potential function of KHDC1L in HNSCC. (a) *KHDC1L* was overexpressed in HNSCC tissues compared with corresponding normal tissues in TCGA. (b) *KHDC1L* was highly expressed in four subtypes (atypical, basal, classical, and mesenchymal) of HNSCC in TCGA. (c) RT-PCR verified the mRNA expression of *KHDC1L* in HOK, SCC9 and CAL27 cell lines. (d–f). GSEA revealed in the *KHDC1L* high expression group proteasome was the most enriched pathway (d), while in the *KHDC1L* low expression group, apoptosis was the maximum enrichment pathway (e), and P53 pathway was also enriched (f). Gene sets with absolute value of normalized enrichment score (|NES|) > 1 and nominal *P*-value (NOM *P*-val) <0.05 were considered significant. Data were presented as the mean ± SD of at least three independent experiments and were analysed with one-way ANOVA (*P < 0.05, ***P < 0.001). TCGA, The Cancer Genome Atlas; GSEA, Gene Set Enrichment Analysis.

To explore the expression level of *KHDC1L* in HNSCC, analysis of the TCGA data via GEPIA (Gene Expression Profiling Interactive Analysis) database **[**16**]** showed *KHDC1L* was aberrantly highly expressed in HNSCC compared with corresponding normal tissues (Figure 1a). Furthermore, in comparison with normal tissues, our subtype analysis of GEPIA revealed that the expression level of *KHDC1*L was elevated in the atypical, basal, classical, and mesenchymal subtypes (Figure 1b). Consistent with the bioinformatics results, RT-PCR experiments verified that expression of *KHDC1L* was higher in human oral cancer cells (SCC9, CAL27) than human oral epithelial cell (HOK) (Figure 1c, *P* < 0.05). To further evaluate the potential biological functions of KHDC1L in HNSCC, we completed the analysis of GSEA (Gene Set Enrichment Analysis) between low expression and high expression of KHDC1L in HNSCC via TCGA. The results indicated that proteasome and apoptosis signal pathways ranked as the most significantly enrichment pathways in the high and low expression of KHDC1L group, respectively (Figure 1d, e) (NOM *P* < 0.05). Additionally, as generally considered to induce apoptosis of tumour cell, P53 pathway was also enriched in the low expression of KHDC1L group (figure 1f) (NOM *P* < 0.05). In summary, all the foregoing results pointed to KHDC1L’s potential role in tumour survival and death especially through apoptosis and P53 pathway in HNSCC, with further biological experimental validation required.

### KHDC1L promotes proliferation in HNSCC cell

**Figure 2.**
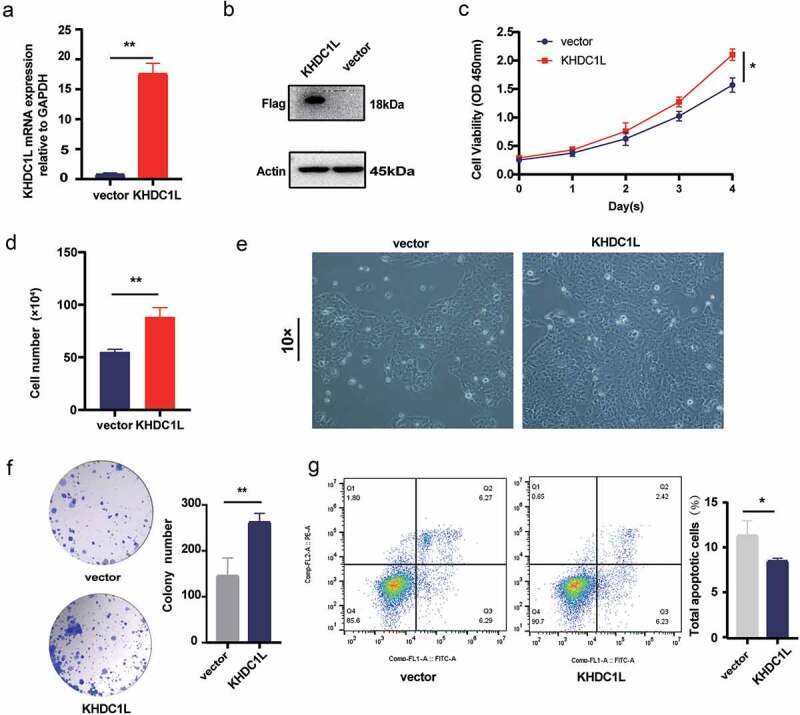
KHDC1L promotes proliferation and inhibits apoptosis in HNSCC cell. (a, b). RT-PCR (a) and western bolt (b) analyses on KHDC1L expression levels in vector and KHDC1L overexpressing group of CAL27 cells. (c). CCK-8 assays of vector and KHDC1L overexpressing group of CAL27 cells. (d). Cell counting in the vector group and KHDC1L overexpression group. (e). Cell morphological change after transfection pcDNA3.1 and pcDNA3.1-KHDC1L. (f). Results of colony formation assays. (g). Flow cytometry determined the percentage of apoptotic cells after overexpressing KHDC1L. Data were presented as the mean ± SD of at least three independent experiments and were analysed with Student’s *t* test or two-way ANOVA (*P < 0.05, **P < 0.01, ***P < 0.001).

To investigate the potential biological functions of KHDC1L in HNSCC cells, CAL27 cell was selected for the following assays. Furthermore, the pcDNA3.1 plasmid and pcDNA3.1-KHDC1L plasmid were constructed in order to upregulate KHDC1L. Since commercial antibody of KHDC1L is unavailable, a sequence of 3× FLAG tag was inserted into the 3’-end CDS region of KHDC1L and recombined with the pcDNA3.1 vector plasmid to detect the protein overexpression level of KHDC1L. As shown in Figure 2a and b, the results of RT-PCR together with western blot demonstrated that the plasmid could effectively enhance the mRNA (*P* < 0.01) and protein expression level of KHDC1L

In addition, cell viability was examined by CCK8, with cell counting and colony formation assays to decide the effect of KHDC1L on proliferation. As shown in Figure 2c, when comparing with the negative control group (*P* < 0.05), overexpression of KHDC1L resulted in a high absorbance at 450 nm on d 4 after transfection. On the basis of the cell counting assay, cell numbers increased on d 4 after transfection in overexpression of KHDC1L group (Figure 2d, *P* < 0.01). In the meantime, we found increasing colony numbers after upregulating KHDC1L (Figure 2f, *P* < 0.01) based on the colony formation assay. In conclusion, KHDC1L elevated the cell viability of CAL27 cell.

### KHDC1L inhibits apoptosis in HNSCC cell

According to the above bioinformatics analysis, apoptotic pathway was evidently enriched in patients with low expression of KHDC1L. Therefore, this study further explored the impact of gene on apoptosis. By observing cell morphological change after transfecting 3 d, we found KHDC1L overexpression could lead to decreasing cell detachment and death (Figure 2e). On the basis of apoptosis detection via flow cytometry, the apoptosis level was significantly diminished after KHDC1L overexpression (Figure 2g, *P* < 0.05), which pointed to KHDC1L’s inhibitory effect on apoptosis in HNSCC cell.

### Transcriptome sequencing analysis on downstream signals of KHDC1L

**Figure 3.**
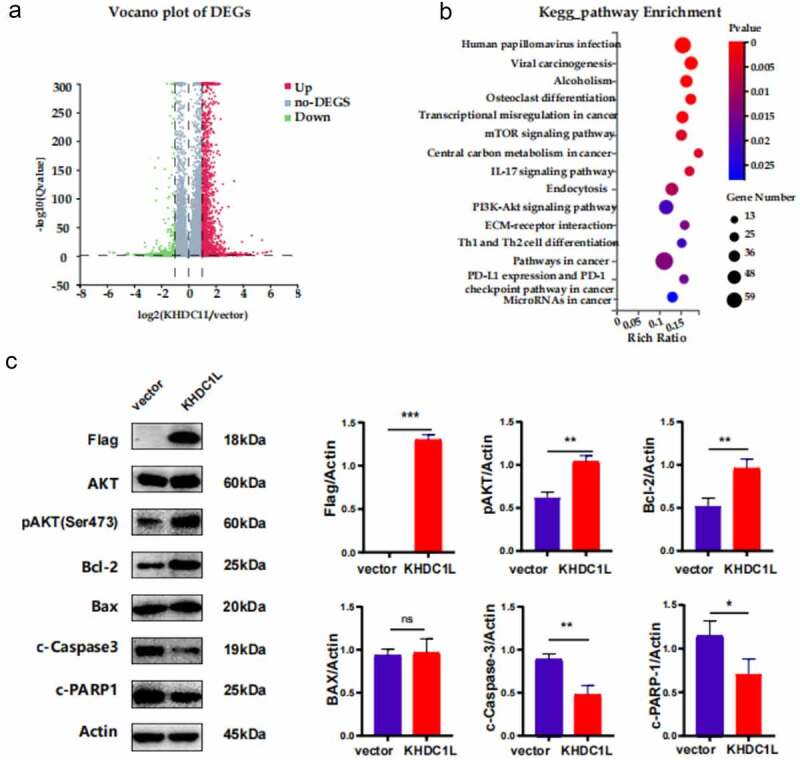
Transcriptome sequencing analysis on downstream signals of KHDC1L. (a). Volcano plot of DEGs. (b). KEGG pathway enrichment of DEGs. (c). Western blot revealed the protein level of Flag, pAKT (Ser473), Bcl-2 were upregulated, and c-Caspase-3 and c-PARP-1 were downregulated. Data were presented as the mean ± SD of at least three independent experiments and were analysed with Student’s *t* test (*P < 0.05, **P < 0.01, ***P < 0.001). DEGs Differentially Expressed Genes; KEGG, Kyoto Encyclopaedia of Genes and Genomes.

Transcriptome sequencing was performed on both overexpression group and negative control group of KHDC1L to determine the downstream signals associated with KHDC1L in CAL27. A total of 1,602 differentially expressed genes (DEGs) were screened taking a threshold of |Log2FC| ≥ 1 and *q* value ≤0.001 (Figure 3a). Subsequently, KEGG results (Figure 3b) figured out enrichment pathways consisting of HPV infection, viral carcinogenesis, alcoholism, osteoclast differentiation, transcriptional misregulation in cancer, mTOR and PI3K-AKT signal pathway, as well as other cancer-related pathways. In accordance with GSEA, pathway in cancer and thyroid cancer pathways were enriched in the KHDC1L overexpression group (Supplementary Figure 1A, 1B). Additionally, GO functional enrichment analysis was realized for DEGs with *q* value ≤0.05 as the cut-off value, indicating enriched biological processes composing of RNA polymerase II-mediated transcriptional regulation, cellular protein metabolic processes, AKT signalling pathway, RhO protein signalling pathway together with apoptosis (Table 1). Finally, protein–protein interaction (PPI) networks of the GO functional enrichment analysis on positive regulation of transcription by RNA polymerase II and apoptosis pathway were constructed, then MYC and HRAS were identified as key genes, respectively (Supplementary Figure 1C, 1D). Both GO and KEGG analyses obviously suggested an abnormal enrichment of AKT signal pathway, leading to an amplified malignant phenotype, as frequently activated in HNSCC. Therefore, it remains to be verified whether AKT serves as the downstream pathway of KHDC1L in CAL27 cell or not.

**Table 1.** GO functional enrichment analysis on the downstream pathways genes of KHDC1L.

GO classification	Enrichment term	Count	*q* Value
Biological Processes (BP)	Positive regulation of transcription by RNA polymerase II	154	8.88e-4
	Positive regulation of transcription, DNA- templated	96	1.25e-3
	Cellular protein metabolic process	41	9.67e-3
	Protein kinase B (AKT) signalling	12	0.02
	Rho protein signal transduction	18	0.02
	Apoptotic process	90	0.03
Molecular Function (MF)	Protein binding	1007	2e-7
	DNA binding	275	7.44e-6
	DNA-binding transcription factor activity, RNA polymerase II-specific	125	7.40e-5
	RNA polymerase II proximal promoter sequence-specific DNA binding	98	1.87e-4
	Transcription factor binding	59	3.81e-3
	Transcription corepressor activity	42	9.04e-3
Cellular Component (CC)	Nucleus	645	1.67e-5
	Nucleoplasm	393	2.04e-4
	Extracellular exosome	243	2.52e-4
	Stress fibre	20	6.38e-4
	Cytoplasm	642	5.98e-3
	Focal adhesion	60	7.14e-3

### Overexpression of KHDC1L activates the AKT and Bcl-2 signal pathways in HNSCC cell

Cell proliferation and apoptosis are regulated by various cell survival-related genes and kinases. On the basis of previous studies, Bcl-2/BAX expression level could affect cell survival, and a decrease in the ratio could induce mitochondrial apoptosis, resulting in an increased cleavage of Caspase-3 (c-Caspase-3) and the substrate cleavage of PARP-1 (c-PARP-1). This study further witnessed an increasing Bcl-2/BAX protein expression ratio as well as decreasing c-Caspase-3 and c-PARP-1 expression level in the KHDC1L overexpression group (Figure 3c).

Based on our results of transcriptome sequencing, it was hypothesized that KHDC1L might activate AKT. Finally, western blot assays verified that overexpression of KHDC1L could enhance the phosphorylation level of AKT at Ser473 site leading to activating signal, which might elevate the expression of Bcl-2 and thus suppress mitochondrial apoptosis (Figure 3c).

### *Pan-cancer analysis on* KHDC1L

**Figure 4.**
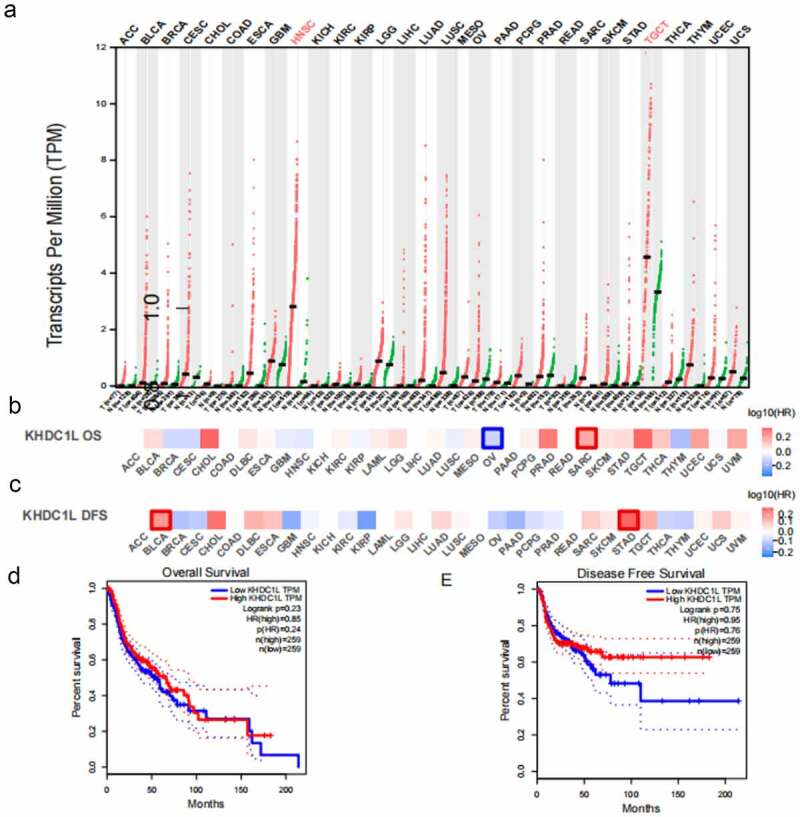
Pan-cancer analysis on KHDC1L. (a). Expression of *KHDC1L* in tumours and corresponding normal tissues in TCGA unveiled the significant upregulation of *KHDC1L* in HNSCC and TGCT. (b). The correlation between KHDC1L expression and log_10_ (HR) of OS in cancers showed negative correlation in SARC and positive correlation in OV. (c). The association between KHDC1L expression and log_10_ (HR) of DFS in cancers showed negative association in BLCA and STAD. (d, e). Kaplan–Meier OS (d) and DFS (e) curves for patients assigned to high and low KHDC1L expression group in HNSCC. TGCT Testicular Germ Cell Tumours; HR, hazard ratio; OS, overall survival; SARC, sarcoma; OV, ovarian serous cystadenocarcinoma; DFS, disease-free survival; BLCA, bladder urothelial carcinoma; STAD, stomach adenocarcinoma.

Hereby, we performed a pan-cancer analysis on KHDC1L through GEPIA database, to find *KHDC1L* expression substantially elevated in HNSCC and Testicular Germ Cell Tumours (TGCT) (Figure 4a). In addition, patients with high-level KHDC1L generally possessed shortened overall survival (OS) in sarcoma (SARC), while a longer OS in ovarian serous cystadenocarcinoma (OV). With regard to disease-free survival (DFS), patients with high-level KHDC1L witnessed shortened DFS in bladder urothelial carcinoma (BLCA) and stomach adenocarcinoma (STAD) (Figure 4b, c). As for HNSCC, the expression of KHDC1L was statistically insignificant for survival (Figure 4d, e).

## Discussion

In accordance with previous studies, RBPs are regarded as crucial regulators for cell survival and differentiation by controlling the post-transcriptional processes of target RNAs [17]. The Human Protein Atlas [18] showed that KHDC1L mRNA was overexpressed in human testis, placenta, lung, and brain tissues but low or absent in other tissues, and single-cell clustering revealed that KHDC1L was enriched in sperm cells. Furthermore, the expression of various RBPs present a tissue-specific way in general, for example, RBM24 was highly expressed in heart tissue and involved in embryonic heart development through regulating the differentiation of embryonic stem cells into cardiomyocytes [19]. Similarly, the tissue-specific distribution of KHDC1L expression may indicate its potential physiological functions, such as spermatogenesis. As shown in a previous study, upregulated KHDC1L was found to be correlated with osteoarthritis and managed to promote synovial cell proliferation, while its expression and function in cancer remained entirely unclear. Our study confirmed that KHDC1L was significantly overexpressed in HNSCC tissues and oral cancer cells (SCC9, CAL27), and revealed its corresponding function in promoting cell proliferation and anti-apoptosis in HNSCC. Moreover, the study demonstrated that AKT and Bcl-2 were KHDC1L downstream signal pathways.

In recent years, in-depth studies on RBPs have figured out their relationship with the malignant phenotype of HNSCC. For instance, as a ‘writer’ of m6A modified way, RBM15 could facilitate proliferation and migration of laryngeal carcinoma cells by modifying TMBIM6 [20] in laryngeal carcinoma. Furthermore, RBM6 is downregulated in laryngeal carcinoma tissues and cell lines, whose upregulation can inhibit cell proliferation and promote apoptosis by activating the ERK pathway [21]. Regarding our study, KHDC1L was overexpressed in HNSCC and TGCT, with GSEA analysis indicating the apoptosis and P53 pathway more active in the low-expressing KHDC1L group. Therefore, our findings implied KHDC1L might be closely associated with cell survival in HNSCC. Excitingly, further functional assays validated the hypothesis that KHDC1L could promote cell viability and inhibit apoptosis in CAL27, distinguishing as a promising specific target for the diagnosis and treatment of HNSCC.

Furthermore, the balance of Bcl-2 and Bax controlling the outer mitochondrial membrane potential could affect the sensitivity to apoptotic signals. This study probed the apoptotic pathway related to KHDC1L, to find increasing Bcl-2/Bax expression, decreasing c-Caspase-3 and c-PARP-1 after overexpression of KHDC1L. Mechanistically, an elevated Bcl-2 binds to Bax forming a heterodimer, which weakens the permeation of mitochondrial membrane, resulting in an anti-apoptotic effect on cells. However, overexpressed KHDC1A induces endoplasmic reticulum-dependent apoptosis in human cervical cancer cells (Hela) in vitro, and induces apoptosis independent of the Bcl-2 pathway in 293 T cells and mouse T cells in vivo [13,14]. Therefore, the complex relationship between KHDC1 family and apoptosis, diverse functions and regulatory pathways may be determined by the distinct structures, cellular localization, and cytogenetic backgrounds. Furthermore, Bcl-2 is clinically related to a poor tumour prognosis, and reduced expression of Bcl-2 sensitizes tumour cells to anti-cancer drugs and radiotherapy. Thus, therapy in Bcl-2-mediated resistance to radiotherapy or chemotherapy, via targeting KHDC1L to inhibit Bcl-2, may be a novel strategy in HNSCC.

In this study, differential genes from the transcriptome sequencing were significantly enriched in the AKT pathway. AKT is the most frequently altered pathway in HNSCC, and aberrant activation could promote proliferation, metastasis and inhibit apoptosis [22]. Further experiment found pAKT (Ser473)/AKT upregulated in the KHDC1L overexpression group, implying an activated AKT signal pathway. A variety of cell survival-related molecules, including the Bcl-2 family, were targets of AKT. For instance, in lymphoma, LINK-A lncRNA was proved to overcome ibrutinib resistance through Akt/Bcl2 pathway [23]. In oral cancer, the silence of AKT1 and AKT2 inhibited the expression of COX-2, cyclinD1, and Bcl-2, which in turn inhibited cell survival [24]. Our study hypothesized that activation of AKT might be an upstream regulator of Bcl-2 expression, but further rescue experiment is needed to verify relevance. Moreover, we also noted an evident enrichment in GO annotations for nuclear components, transcriptional processes, DNA binding, and transcription factor binding entries for KHDC1L (a cytoplasmic protein), thus speculating that KHDC1L might also directly involve in the transcriptional regulation of downstream target genes (e.g., Bcl-2) by regulating transcription factors.

Although AKT pathway is activated in various cancers, some eventually escape the pathway inhibitior due to compensation, leaving therapies targeting on this pathway remain unsatisfactory. Therefore, seeking new targets against AKT pathway holds great significance. Fortunately, KHDC1L was found to regulate both AKT and Bcl-2 in HNSCC, and it is reasonable to excavate the potential for KHDC1L to be a new therapeutic target.

Overall, this study firstly explored the biological function of KHDC1L in HNSCC. KHDC1L was overexpressed in HNSCC, promoted proliferation, and inhibited apoptosis in CAL27 cell. Moreover, overexpression of KHDC1L in CAL27 cell activated the downstream signalling AKT and Bcl-2.

## Supplementary Material

Supplemental Material

## Data Availability

All bioinformatics analyses in this study were performed by means of the online portal GEPIA (http://gepia.cancer-pku.cn/detail.php), with original data available on public database of TCGA (https://portal.gdc.cancer.gov/). The RNA-seq and biological experimental data involved in this manuscript are available, and we promise to upload all data to the specified database as required by this journal when accepted (http://gepia.cancer-pku.cn/detail.php?gene=KHDC1L).
