# Supplementary figures and images for: Upregulation of KHDC1L promotes the proliferation and inhibits apoptosis in head and neck squamous cell carcinoma
Source: Epigenetics. 2023 Feb 3;18(1):2175168. doi: 10.1080/15592294.2023.2175168 (PMC9980683; doi:10.1080/15592294.2023.2175168)

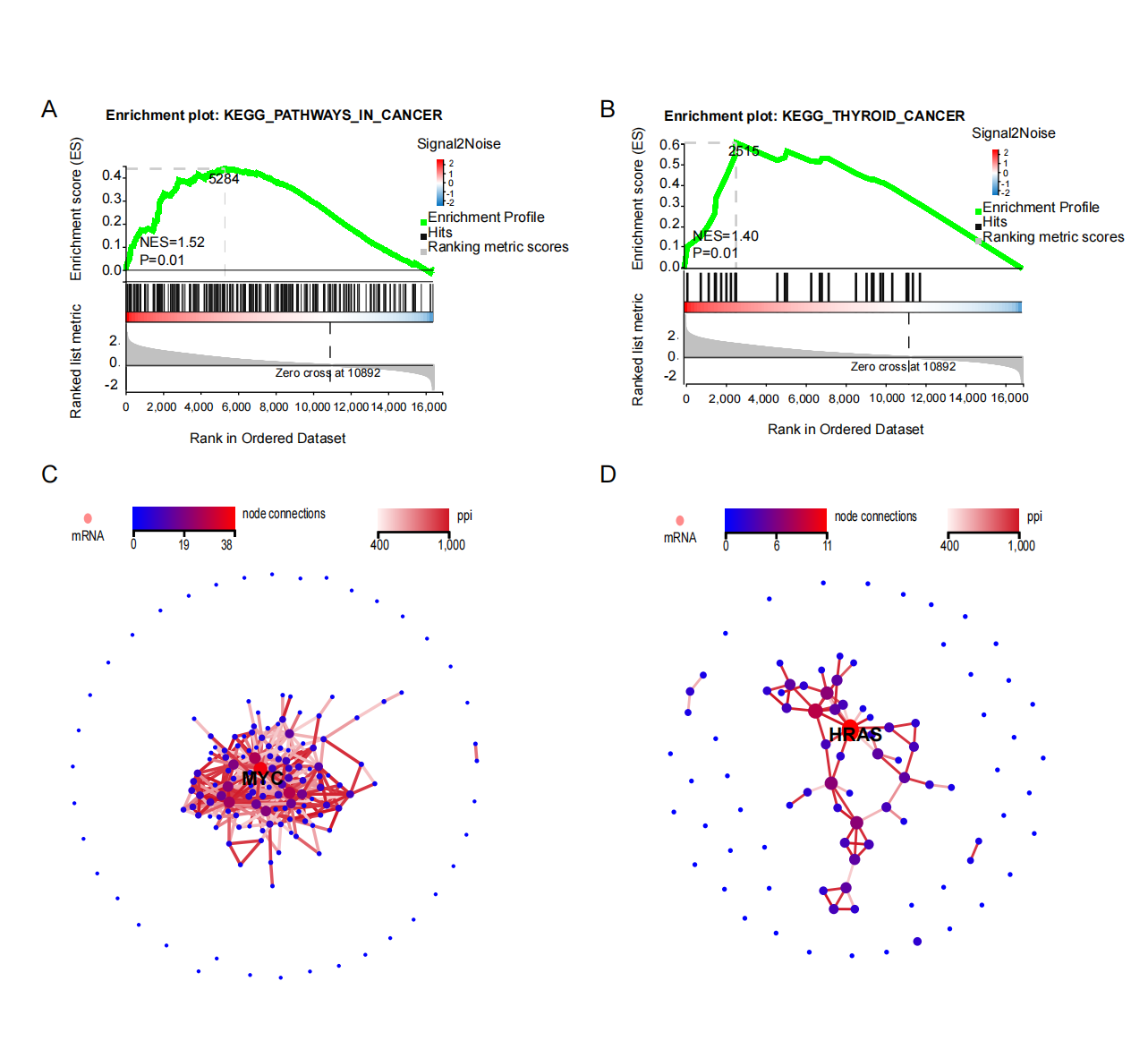

Supplement: Supplemental Material [file KEPI_A_2175168_SM4742.tif]
